# Analysis of vertical deficiency in infraoccluded primary molars: an investigation of dental, gingival, and alveolar parameters

**DOI:** 10.1007/s10266-025-01306-y

**Published:** 2026-01-14

**Authors:** Teresa Temming, Lisa Bondarjanska, Robert Strenger, Anahita Jablonski-Momeni, Heike Korbmacher-Steiner

**Affiliations:** Department of Orthodontics, Clinic of Dentistry, Philipps-Universität Marburg, Georg-Voigt-Str. 3, 35039 Marburg, Germany

**Keywords:** Primary molar, Infraocclusion, Gingival margin, Alveolar ridge, Vertical deficiency

## Abstract

The study aims to evaluate the vertical deficiency associated with infraoccluded primary molars by quantitatively analyzing dental, gingival, and alveolar parameters, and to determine their potential diagnostic and therapeutic relevance in clinical practice. A total of 290 infraoccluded primary molars from 129 patients under 30 years of age were retrospectively analyzed (data collection between 2000 and 2023). Inclusion required at least one infraoccluded primary molar (> 0.5 mm), along with diagnostic dental casts and panoramic radiographs taken within six months. Parameters evaluated included the degree of infraocclusion, gingival margin levels, and alveolar ridge morphology. The presence or absence of the successor tooth was considered. The mean vertical displacement of infraoccluded molars was 1.6 mm (SD 0.87). Atypical gingival margins were observed in 35% (*n* = 122) of infraoccluded molars, compared to 4% (*n* = 2) in controls, with higher prevalence in the mandible and second primary molars. The frequency of atypical gingival margins and alveolar ridge morphologies increased with the severity of infraocclusion (*P* < 0.05, respectively). An infraocclusion of ≥ 2.0 mm predicted atypical alveolar morphology with 70.6% sensitivity and 87.6% specificity. A significant association was also observed between atypical alveolar ridges and atypical gingival margins (*P* < 0.05). Multiple regression analysis (adjusted R^2^ = 0.42) revealed that infraocclusion severity was significantly associated with age, sex, tooth type, gingival margin, and alveolar ridge morphology, whereas agenesis of the permanent successor showed no significant association. Infraocclusion of primary molars influences not only occlusal relationships but also gingival and alveolar morphology. Atypical gingival and alveolar ridge patterns may offer valuable supplementary diagnostic criteria for the management of dental ankylosis. Further prospective studies are needed to investigate their potential for use in clinical decision-making.

## Introduction

Infraoccluded primary molars represent a relevant focus in orthodontics and pediatric dentistry due to their high prevalence and potential influence on children's alveolar and dental development [1–4]. Prevalence rates reported in the literature vary, documenting a rate of up to 22.8% in the general population and 32.7% in twins [2]. Despite extensive research, precise knowledge of the etiology of infraocclusion remains limited. While earlier studies emphasized local factors—such as trauma or localized metabolic disturbances—recent evidence increasingly points to endogenous factors [5, 6]. Infraoccluded primary molars are characterized by ankylosis, a pathological fusion of the root cementum or dentin to the alveolar bone [7]. This fusion may be localized and is subject to osteoclastic resorption, allowing for possible physiological exfoliation. However, when ankylosis extends—particularly in the furcation area—it may inhibit periodontal bone growth and contribute to progressive infraocclusion, especially during periods of active skeletal development [8, 9].

Clinically, infraoccluded primary molars exhibit a distinct, high-pitched percussion sound, lack of mobility, and an occlusal discrepancy relative to adjacent teeth [10]. Infraocclusion has been widely measured [11–14], but the variability in reference levels limits comparability between studies. To date, no studies have systematically examined gingival morphology associated with infraocclusion. Alveolar changes—such as vertical bone loss at the enamel–cementum junction or a concave ridge contour—may indicate disrupted alveolar development, yet few studies have explored these aspects in depth [8, 15, 16].

Earlier studies classified primary tooth ankylosis according to various criteria, including time of onset, extent of infraocclusion, tilting of adjacent teeth, degree of root resorption, and agenesis of the permanent successor [17–19]. These parameters have formed the basis for therapeutic decisions to date. However, it was shown that the tilting of adjacent teeth is primarily caused by diverging roots with converging crowns, which has only a minor impact on the available occlusal space [20, 21]. Furthermore, root resorption of infraoccluded primary molars seems to play a minor role compared to the extent of infraocclusion, and that primary molars with short roots and no successor can remain in situ for a prolonged time [12, 22, 23].

The objective of this study was therefore to thoroughly investigate the vertical deficiency associated with infraoccluded primary molars through quantitative assessment in relation to the presence or absence of the permanent successor. By evaluating dental, gingival, and alveolar parameters, this study aimed to identify clinically relevant correlations and explore their diagnostic and therapeutic implications.

## Material and methods

### Study design, cohort, and general conditions

This retrospective study included 129 patients with infraoccluded primary molars referred to the Department of Orthodontics, University of Marburg. Data were collected from 01.01.2000 to 31.12.2023. Ethical approval was obtained from the responsible Ethics Committee of the Faculty of Medicine (Ref. No. 23-239 RS). Inclusion criteria were: age below 30 years, presence of at least one primary molar in infraocclusion (≥ 0.5 mm), and availability of at least one diagnostic dental cast and a corresponding panoramic radiograph taken within a 6-month interval. All available diagnostic models and panoramic radiographs were analyzed. Patient data were anonymized using Microsoft^®^ Excel^®^ 2021. Dental casts were digitized using the iTero Lumina™ intraoral scanner (V 5.0, Align Technology, San Jose, CA, USA) and evaluated using Blender software (V 4.2, Blender Foundation, Amsterdam, Netherlands). Panoramic radiographs were analyzed digitally (Adobe Photoshop V 25.0, Adobe Inc., San Jose, CA, USA), while analog panoramic radiographs were analyzed manually. Both maxillary and mandibular arches were included.

### Examiner calibration

Data were collected by two examiners (RS, LB), following a comprehensive training and calibration process led by two experienced orthodontists (TT, HKS). In cases of uncertainty, a consensus decision was reached through discussion. Intra-rater and inter-rater reliability for identifying infraoccluded primary molars, measuring infraocclusion, assessing the gingival margin, and classifying alveolar ridge morphology showed very good to perfect agreement (Cohen’s Kappa = 0.8 to 1.0).

### Assessment

**Fig. 1:**
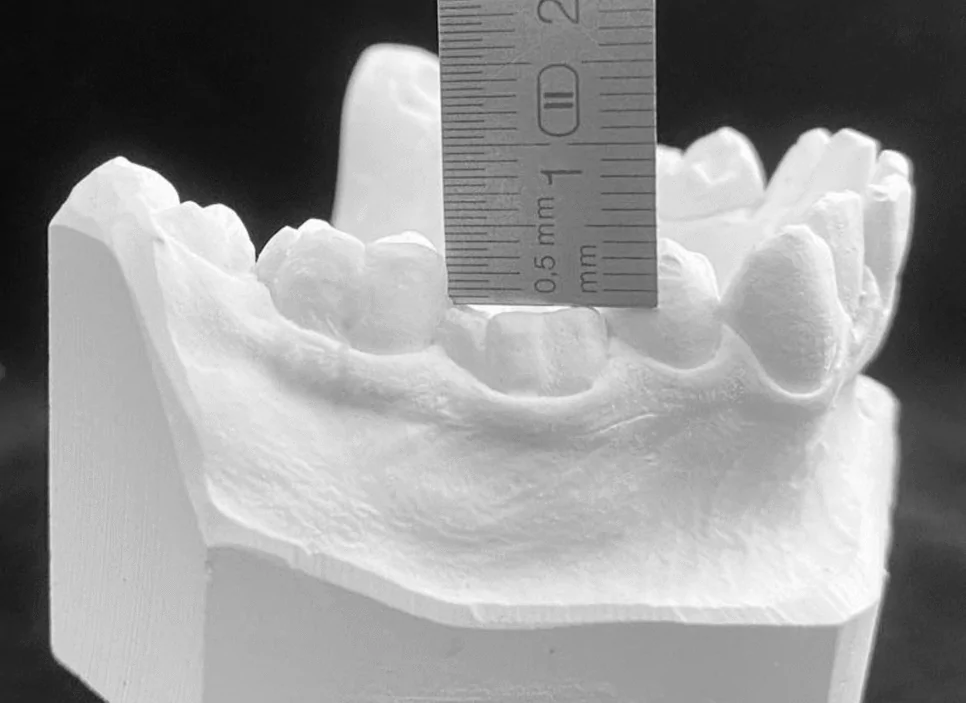
Measurement of infraocclusion as the vertical distance between the distal marginal ridge of the infraoccluded primary molar and the mesial marginal ridge of the adjacent permanent first molar (accuracy: 0.5 mm)

**Fig. 2:**
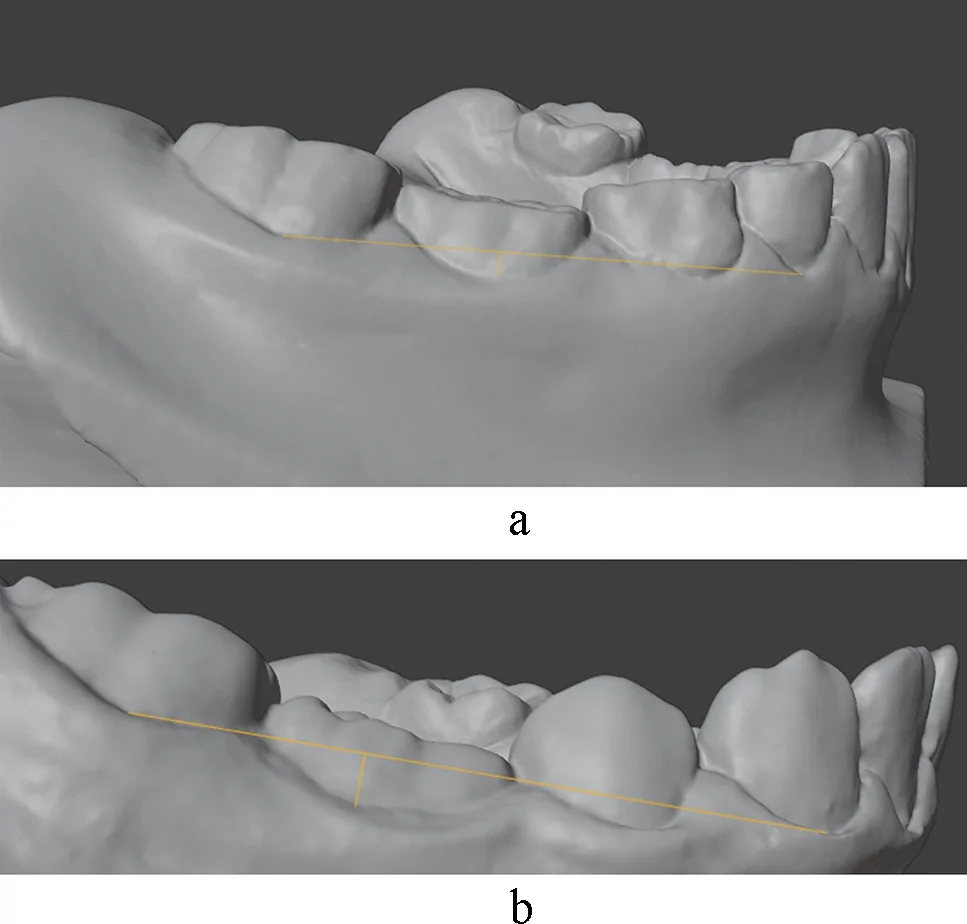
**A** Measurement of the gingival margin as the distance from the lowest point of the gingival margin of the infraoccluded primary molar to the line connecting the deepest gingival margins of the adjacent permanent first molar and the primary canine. **B** Measurement of the gingival margin using the permanent canine as the reference point (accuracy: 0.1 mm)

**Fig. 3:**
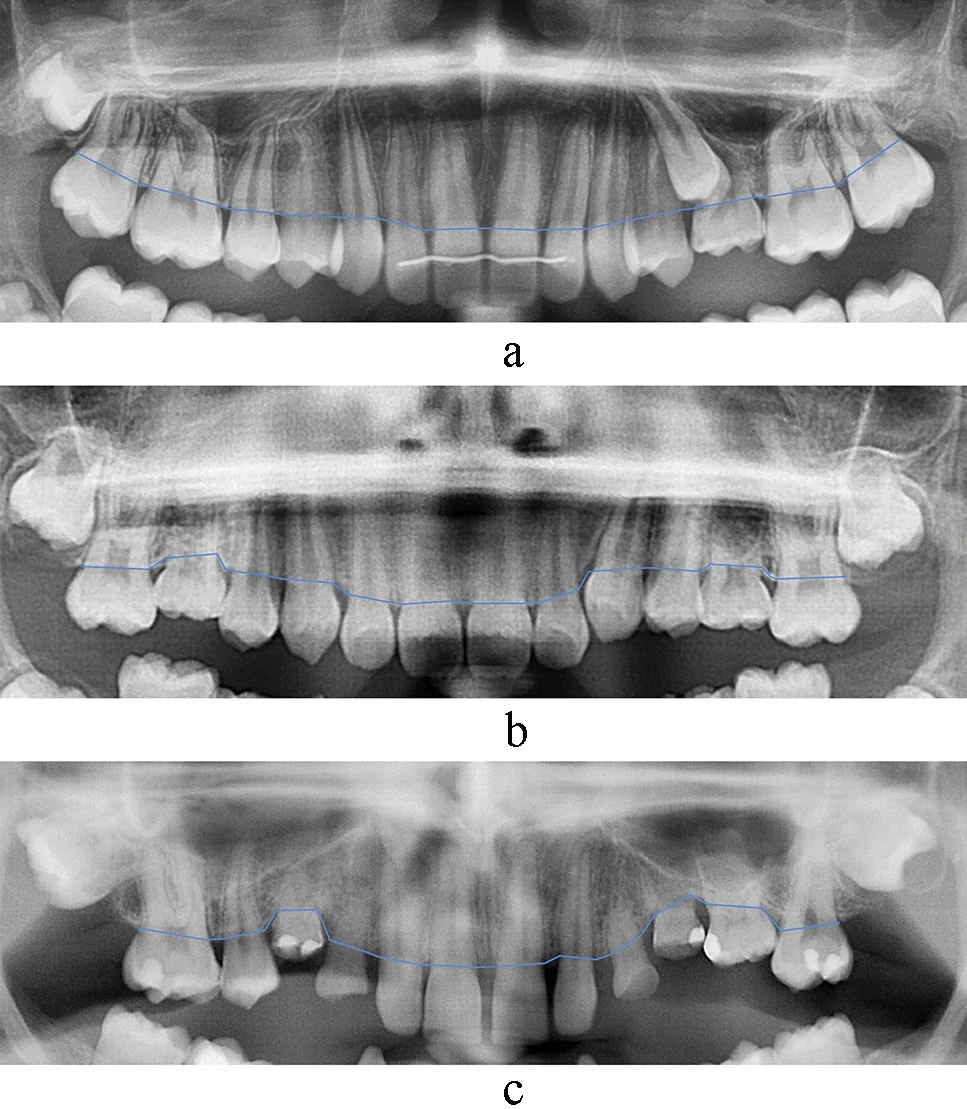
Radiographic assessment of alveolar ridge morphology—**A** horizontal, **B** vertical, **C** concave

- Infraocclusion: assessed on analog dental casts following the method of Shalish et al. [14] (Fig. 1). In cases where the permanent first molar was absent, the second primary molar was used as a reference point. If both the permanent first molar and the second primary molar were missing, the measurement was considered not applicable. The severity of infraocclusion was classified according to the criteria by Odeh et al. [13] (Table 1).
- Gingival margin: measured on digitized dental casts. The vertical distance from the deepest point of the gingival margin of an infraoccluded molar to a defined reference plane was evaluated (Fig. 2A and B). The reference plane was established by connecting the deepest points of the gingival margins of the adjacent permanent first molar and the primary or permanent canine. If these reference teeth were absent or erupting, measurement was not feasible. In patients with a healthy contralateral molar and according reference teeth in situ, corresponding measurements were recorded for control comparison.
- Alveolar ridge morphology: assessed according to Dias et al. [24] by delineating the crestal line at the enamel–cementum junction across the arch (Fig. 3A–C). If the successor premolar was erupting through the alveolar crest, analysis was excluded. Alveolar ridge morphology was classified as horizontal, vertical, or concave (Table 1).
- Tooth Agenesis: The presence of a permanent successor to an infraoccluded first or second primary molar was evaluated. Agenesis of second premolars was only evaluated in cases where a panoramic radiograph taken at ≥ 10 years of age was available.

**Table 1:** Assessment of infraoccluded primary molars on three levels—dental, gingival, and alveolar

**Infraocclusion**	
Mild:	0.5 mm to < 2 mm
Moderate:	2 mm to < 3 mm
Severe:	≥ 3 mm
**Gingiva margin**	
Typical:	> − 1.41 mm^a^ or > − 0.71 mm^b^ respectively
Atypical:	≤ − 1.41 mma or ≤ − 0.71 mm^b^ respectively
**Alveolar ridge morphology**	
Horizontal:	Physiological pattern^c^
Vertical:	Localized vertical bone recession in the region of the infraoccluded primary molar^d^
Concave:	Radial contour, neighboring teeth affected, the deepest point at the site of the infraoccluded primary molar^d^

^a^Primary canine as reference point, ^b^permanent canine as reference point, ^c^typical alveolar ridge morphology, ^d^atypical alveolar ridge morphology

### Statistical analysis

Statistical analyses were performed using MedCalc^®^ software (version 22.021, MedCalc Software Ltd, Ostend, Belgium). Data were tested for normal distribution using the Shapiro–Wilk’s test (*p* < 0.05). Non-parametric tests were applied if the assumption of normal distribution was violated. Categorical variables were analyzed using the chi-squared test. For ordinal and interval-scaled variables, the Spearman rank correlation coefficient was used. The predictive accuracy of the gingival margin was assessed using receiver operating characteristic (ROC) curve analysis, and cut-off values were determined to categorize gingival margins into typical and atypical patterns. Mann–Whitney U test and Kruskal–Wallis test were used for group comparisons. Multiple regression analysis was conducted to identify the significant factors associated with infraocclusion simultaneously (*P* < 0.05).

## Results

The sample consisted of 129 patients (68 females, 61 males) diagnosed with a total of 290 infraoccluded primary molars. The mean number of infraoccluded primary molars per patient was 2.2 (SD 1.3). In 24 patients, more than one diagnostic dental cast and panoramic radiograph was available, resulting in 160 diagnostic records analyzed. This yielded 344 infraoccluded primary molars for evaluation. The mean age at the time of evaluation was 10.7 years (range 5–26 years). The age distribution was as follows: 42% (*n* = 145) of cases were between 5 and 9 years (early mixed dentition), 47% (*n* = 163) between 10 and 14 years (late mixed dentition), and 11% (*n* = 36) between 15 and 30 years (permanent dentition). 82% (*n* = 283) of the infraoccluded primary molars were located in the mandible, and 64% (*n* = 221) involved second primary molars. In 31.1% (*n* = 107) of cases, agenesis of the successor was observed (Table 2).

**Table 2:** The descriptive data of the infraoccluded primary molars included in the study is shown as a percentage (*n*)

	Tooth								Total
	55	54	64	65	75	74	84	85	
	*n* = 18	*n* = 12	*n* = 11	*n* = 20	*n* = 102	*n* = 52	*n* = 48	*n* = 81	*n* = 344
**Age** (year)									
Mean ± SD	12.8 ± 4.9	10.4 ± 3.4	10.9 ± 2.9	13.2 ± 4.6	11.5 ± 2.7	8.8 ± 2.0	8.0 ± 1.8	11.3 ± 2.6	**10.7 ± 3.2**
**Gender**									
Male	50 (9)	50 (6)	54.5 (6)	60 (12)	44.1 (45)	55.8 (29)	52.1 (25)	42 (43)	**48.3 (166)**
Female	50 (9)	50 (6)	45.5 (5)	40 (8)	55.9 (57)	44.2 (23)	47.9 (23)	58 (47)	**51.7 (178)**
**Successor tooth**									
No absence	44.4 (8)	83.3 (10)	81.8 (9)	55 (11)	51 (52)	100 (52)	95.8 (46)	60.5 (49)	**68.9 (237)**
Absence	55.6 (10)	16.7 (2)	18.2 (2)	45 (9)	49 (50)	0	4.2 (2)	39.5 (32)	**31.1 (107)**
**Infraocclusion**									
Mild	68.7 (11)	75 (9)	36.4 (4)	38.9 (7)	66.3 (63)	57.7 (30)	66 (31)	60.8 (48)	**61.5 (203)**
Moderate	18.8 (3)	16.7 (2)	45.5 (5)	50 (9)	23.2 (22)	30.8 (16)	23.2 (12)	22.8 (18)	**26.4 (87)**
Severe	12.5 (2)	8.3 (1)	28.3 (2)	11.1 (2)	10.5 (10)	11.5 (6)	10.5 (4)	16.5 (13)	**12.1 (40)**
**Gingiva margin**									
Typical	77.8 (14)	100 (12)	81.8 (9)	85.0 (17)	55.9 (57)	64.4 (34)	77.1 (37)	51.9 (42)	**64.5 (222)**
Atypical	22.2 (4)	0	18.2 (2)	15.0 (3)	44.1 (45)	34.6 (18)	22.9 (11)	48.1 (39)	**35.5 (122)**
**Alveolar ridge morphology**									
Horizontal	61.1 (11)	88.9 (8)	75 (6)	66.7 (12)	82.3 (79)	90 (40)	83.7 (36)	87.0 (67)	**82.7 (259)**
Vertical	38.9 (7)	0	12.5 (1)	27.8 (5)	17.7 (17)	9.1 (4)	16.3 (7)	13.0 (10)	**16.3 (51)**
Concave	0	11.1 (1)	12.5 (1)	5.6 (1)	0	0	0	0	**1.0 (3)**

### Infraocclusion

Most infraoccluded primary molars exhibited mild infraocclusion (61.5%, *n* = 203). The mean infraocclusion was 1.6 mm (SD 0.87; range 0.5–4 mm). Bivariate analysis showed no statistically significant association between the degree of infraocclusion and jaw (maxilla vs. mandible; *P* = 0.2934), or tooth type (first vs. second primary molar; *P* = 0.9575). A significant age dependency was also not observed (*P* = 0.1). 

However, multiple regression analysis demonstrated that seven of eight factors examined exhibited a significant association with the severity of infraocclusion (R^2^-adjusted = 0.4203; Table 5). Specifically, infraocclusion increased by an average of 0.06 mm per year. In girls, infraocclusion was on average 0.17 mm more pronounced than in boys. In contrast, infraocclusion was found to be 0.22 mm less pronounced in the mandible, and 0.37 mm less pronounced on average in the second primary molars. Agenesis of the permanent successor was the only factor examined that did not demonstrate a significant association with infraocclusion.

### Gingival margin

**Fig. 4:**
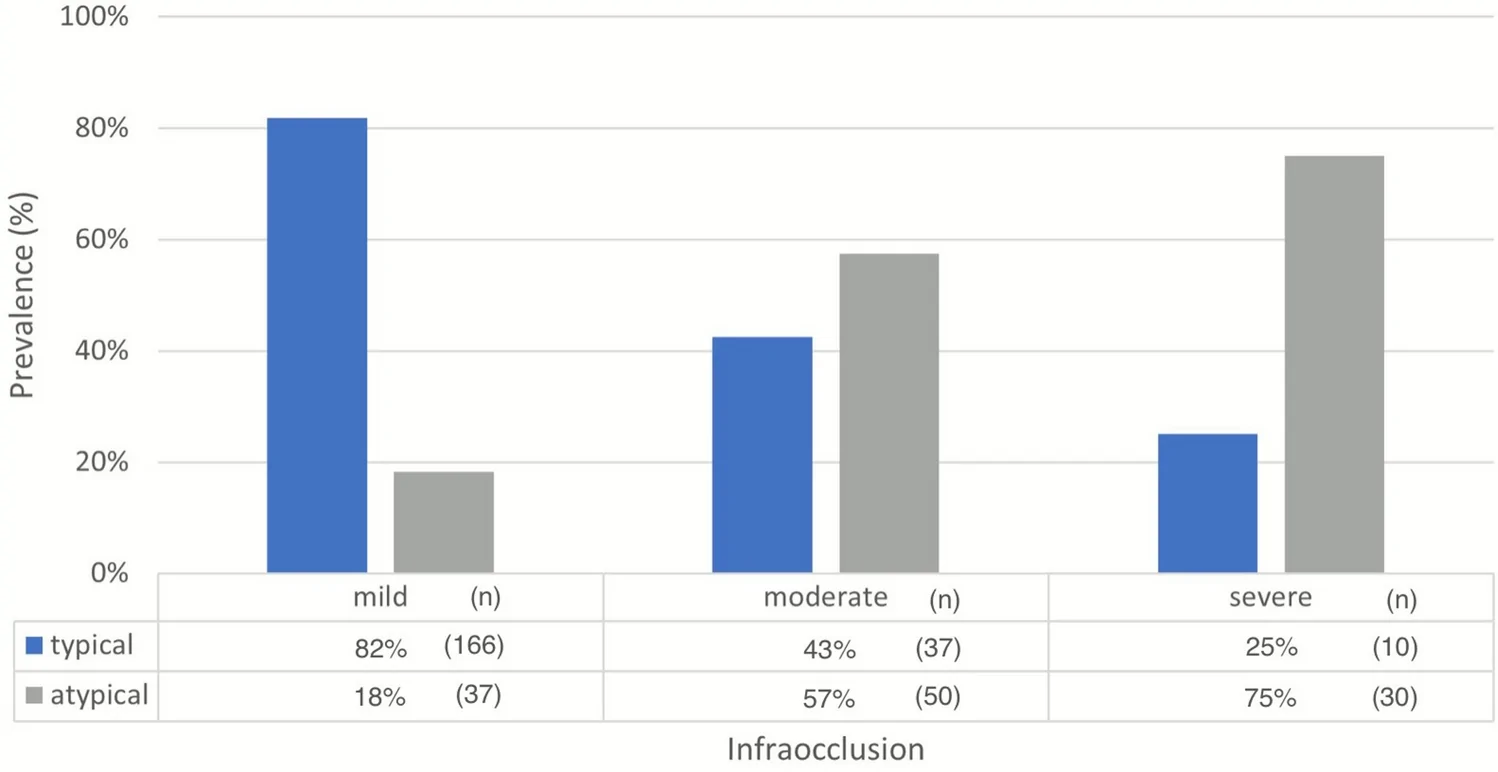
Prevalence of infraocclusion and gingival margin levels in infraoccluded primary molars

In addition to the 344 infraoccluded primary molars, 47 contralateral, healthy primary molars were included for comparison. The mean vertical distance from the gingival margin to the reference plane in healthy primary molars was 0.26 mm (SD = 0.93; range: − 1.3 to 1.7 mm) when the primary canine served as the reference, and 0.73 mm (SD = 1.02; range: − 1.1 to 2.4 mm) when using the permanent canine. For infraoccluded teeth, the corresponding distances were − 0.74 mm (SD = 1.14; range: − 3.8 to 2.8 mm) and − 0.40 mm (SD = 1.33; range: − 3.4 to 3.3 mm), respectively. The ROC curve analysis identified cut-off values of − 1.41 mm (primary canine) and − 0.71 mm (permanent canine) for classification of gingival margin as typical or atypical (Tables 1 and 3). The analysis revealed a significantly higher area under the curve (AUC) when the permanent canine was used as the reference point (*P* < 0.05). Atypical gingival margins were significantly more frequent in infraoccluded primary molars (35%, *n* = 122) compared to healthy controls (4%, *n* = 2; *P* < 0.05). In infraoccluded primary molars, atypical gingival margins were significantly more frequent in mandibular molars than in maxillary molars (*P* < 0.05) and were more prevalent in second primary molars than in first primary molars (*P* < 0.05). In addition, atypical gingival margins were observed more frequently in the early mixed dentition compared to the late mixed or permanent dentition (*P* < 0.05). The frequency of atypical gingival margins increased with the degree of infraocclusion (*P* < 0.05): 18% (*n* = 37) of cases with mild infraocclusion exhibited atypical gingival margins compared to 75% (*n* = 30) in cases of severe infraocclusion (Fig. 4). According to the multiple regression analysis, an atypical gingival margin was associated with a 0.58 mm greater infraocclusion (Table 5).

**Table 3:** ROC curve analysis: Gingival margin in patients with mild versus moderate–severe infraocclusion

Variable	Parameter	Sensitivity ≈ Specificity	AUC (SEM)
Gingival margin assessment using the primary canine as a reference point			
Gingiva margin	Cut-off point	− 1.41 mm	0.692 (0.0430)
	Sensitivity	51.5% (95% CI 39.0–63.8)	
	Specificity	85.1% (95% CI 77.2–91.1)	
Gingival margin assessment using the permanent canine as a reference point			
Gingival margin	Cut-off point	− 0.71 mm	0.804 (0.0369)
	Sensitivity	76.27% (95% CI 63.4–86.4)	
	Specificity	77.53% (95% CI 67.4–85.7)	

AUC: area under curve, CI: confidence interval, SEM: standard error of mean

### Alveolar ridge morphology

Horizontal alveolar ridge morphology was observed in the majority of cases (82.7%, *n* = 250), while a concave ridge form was rare (1%, *n* = 3). Atypical alveolar ridge (vertical, concave) morphology occurred significantly more often in the maxilla (*P* < 0.05). No significant association was found between alveolar ridge morphology and the type of primary molar affected (*P* = 0.24). However, atypical alveolar ridge patterns were significantly more common in the late mixed and permanent dentition compared to the early mixed dentition (*P* < 0.05).

**Fig. 5:**
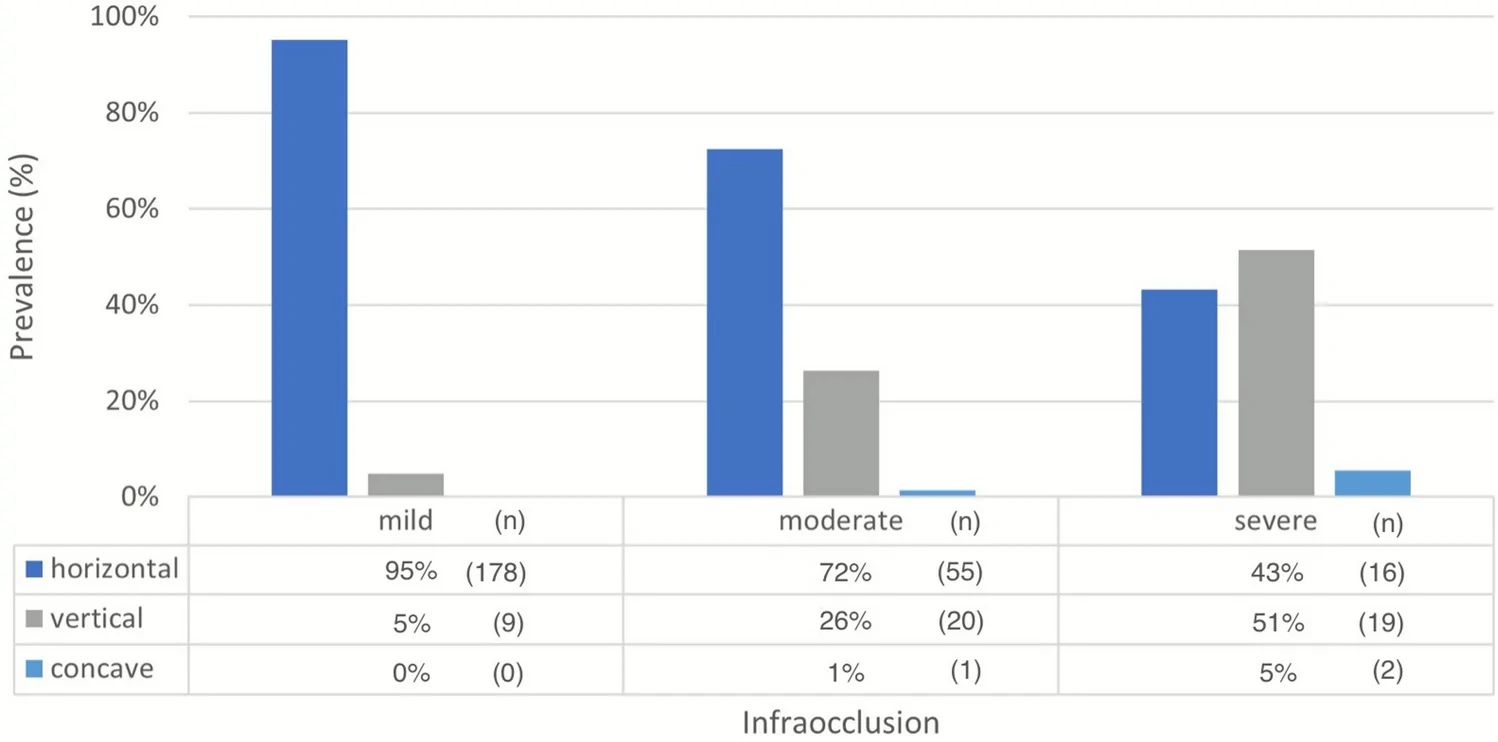
Prevalence of infraocclusion and alveolar ridge morphology in infraoccluded primary molars

**Fig. 6:**
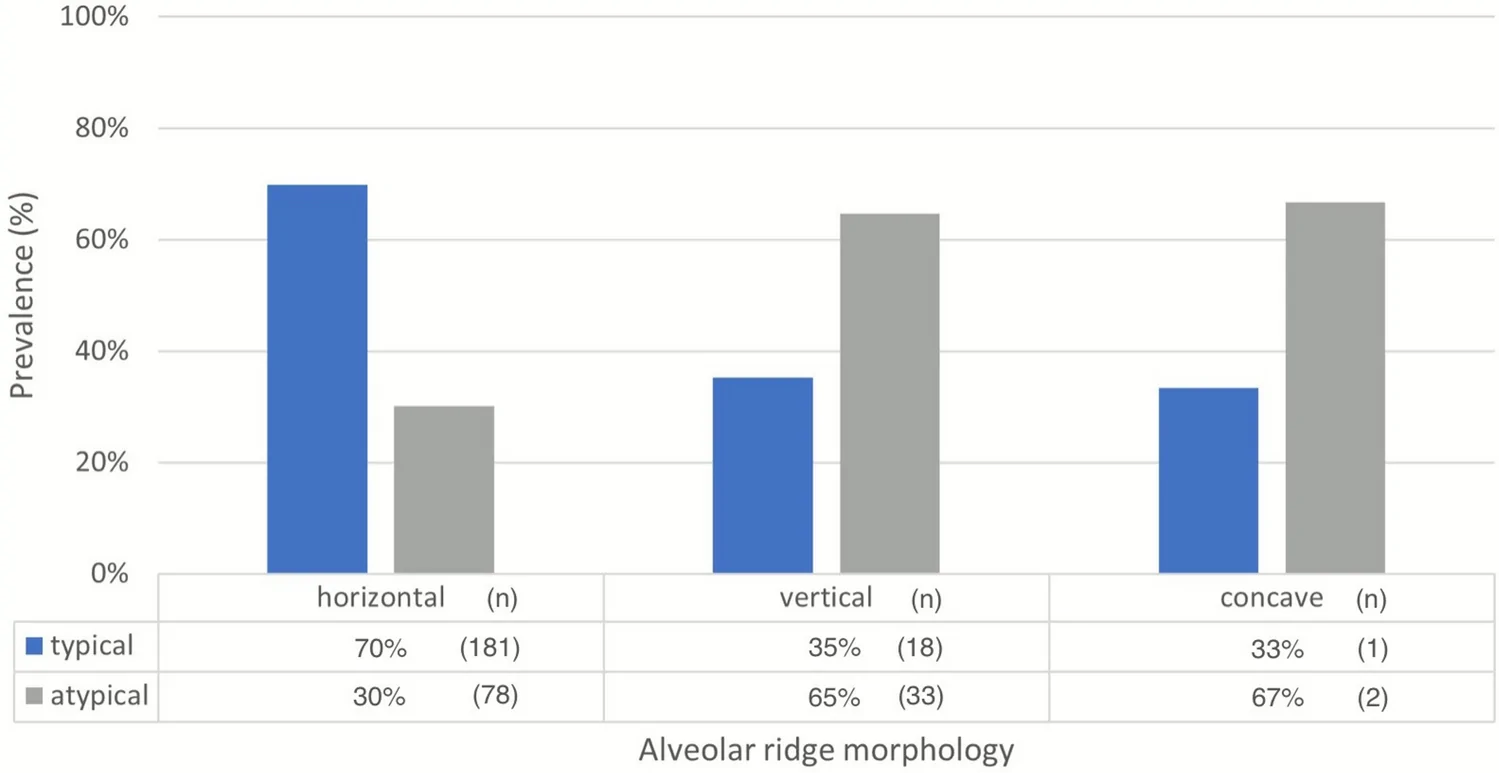
Prevalence of gingival margin levels and alveolar ridge morphology in infraoccluded primary molars

The prevalence of atypical alveolar ridge morphology increased significantly with the severity of infraocclusion (*P* < 0.05). Whereas 95% (*n* = 178) of cases with mild infraocclusion were associated with physiologic ridge morphology, only 43% (*n* = 16) with severe infraocclusion presented a physiologic pattern. The initial observation of a concave alveolar ridge pattern was recorded in one case of moderate infraocclusion (Fig. 5). According to ROC curve analysis, the infraocclusion threshold for predicting an atypical alveolar ridge was 2.0 mm, with a sensitivity of 70.6% and specificity of 87.6% (Table 4). According to the multiple regression analysis, a concave alveolar ridge morphology was associated with a 0.66 mm greater infraocclusion (Table 5). A significant correlation was found between atypical alveolar ridge morphology and atypical gingival margin (*P* < 0.05): 30% (*n* = 78) of cases with horizontal ridge morphology exhibited atypical gingival margins, compared to 65% (*n* = 33) among those with vertical alveolar ridge forms (Fig. 6).

**Table 4:** ROC curve analysis: Infraocclusion in patients with physiological versus atypical alveolar ridge morphology

Variable	Parameter	Sensitivity ≈ Specificity	AUC (SEM)
Infraocclusion	Cut-off point	2 mm	0.840 (0.0315)
	Sensitivity	70.6% (95% CI 56.2–82.5)	
	Specificity	87.6% (95% CI 82.8–91.4)	

AUC: area under curve, CI: confidence interval, SEM: standard error of mean

**Table 5:** Multiple regression analysis of factors associated with infraocclusion (adjusted R^2^ = 0.42)

Predictor variables	Regression coefficient	*P* value
Age	0.0595	0.0002*
Gender	0.1689	0.0346*
Jaw	− 0.2215	0.0520*
Tooth type	− 0.3677	0.0001*
Tooth agenesis of the successor		> 0.05
Gingival margin	0.5780	< 0.0001*
Alveolar ridge morphology	0.6619	< 0.0001*

*Significant association with infraocclusion

## Discussion

Previous studies on infraoccluded primary molars focused primarily on dental aspects, [21, 26–30] with some examining alveolar changes [15, 24, 31, 32]. However, the clinical implications of latter findings have received limited attention in treatment planning and decision-making [17–19]. The present study expands on current knowledge by introducing gingival parameters as additional indicators of the vertical deficiency of infraoccluded primary molars.

### Infraocclusion

The predominance of mild infraocclusion and the comparatively low rate of severe infraocclusion are consistent with previous reports [29]. Bivariate analyses revealed no significant associations between infraocclusion severity and jaw or tooth type. In contrast, multiple regression analysis demonstrated more pronounced infraocclusion in the maxilla, in accordance with findings reported in the literature [29, 32, 33]. Moreover, primary first molars were more severely affected in the multiple regression analysis, whereas Silvestrinit Biaviati et al. [34] reported a greater involvement of second primary molars. The discrepancy between the findings of bivariate and multiple regression analyses highlights the importance of adjusted analyses. This suggests that the observed effects of individual variables may be distorted or modified by other clinical factors.

In the present cohort, agenesis of the permanent successor was observed in 31% of infraoccluded primary molars, representing a substantially higher prevalence than that reported for the general population (0.66–3.62%) [35–37]. Nevertheless, numerous studies have confirmed a strong association between primary tooth ankylosis and hypodontia [14, 21, 38–40]. While recent studies have reported more severe infraocclusion in the absence of a permanent successor [25, 26] this finding could not be confirmed in the present study using multiple regression analysis. The lack of a significant association in the multiple regression model may be due to confounding factors, such as age and tooth-related variables, as well as the heterogeneous and non-linear progression of infraocclusion. As Bjerklin und Bennett [12] previously described, infraocclusion does not follow a consistent temporal pattern. In a retrospective observational design, this may limit the ability to assess the potential influence of successor agenesis at a given time point. To account not only for infraocclusion but also for the widely described growth-inhibiting effect of primary tooth ankylosis [20, 24, 31, 32], the present study therefore incorporated additional gingival and alveolar parameters to more comprehensively characterize the vertical deficit associated with infraoccluded primary molars.

### Gingival margin

To the authors’ knowledge, gingival margin characteristics of infraoccluded primary molars have not been addressed in prior literature. The study demonstrated a significant difference in the gingival contour between infraoccluded primary molars and the control group, both with regard to the frequency of atypical gingival patterns (infraoccluded primary molars: 35%, *n* = 122; controls: 4%, *n* = 2) and the maximum vertical discrepancy (− 3.8 mm versus − 1.3 mm). However, the relatively modest size of the control group must be considered when interpreting these findings.

Several characteristics were associated with atypical gingival margins, including higher prevalence in mandibular teeth, in second primary molars, and with increasing severity of infraocclusion. The increased occurrence in the mandible was unexpected, as previous literature describes pronounced infraocclusion in the maxilla [29, 32, 33]. The increased involvement of second primary molars is consistent with reports in the literature associating these teeth with more advanced stages of infraocclusion [34]. However, this observation contrasts with the present findings, which demonstrated a lower degree of infraocclusion in primary second molars compared with primary first molars.

The permanent canine proved to be a more reliable reference point for gingival margin classification than the primary canine, likely due to its anatomical morphology and the resulting greater predictive accuracy. Notably, ROC curve analysis compared mild infraocclusion to moderate and severe cases, based on the assumption that the inhibitory effect of infraocclusion on alveolar bone increases with its severity [31].

### Alveolar ridge morphology

Atypical alveolar ridge morphology was significantly correlated with an atypical gingival margin. In contrast to the atypical gingival margin, which was found more frequently in the mandible, atypical alveolar ridge patterns were found more frequently in the maxilla. The investigation revealed no statistically significant correlation between tooth type and the alveolar ridge morphology. As reported by Kjaer et al. [31] the frequency of atypical alveolar ridge patterns increased with infraocclusion severity (Fig. 5). Interestingly, concave ridge morphology was observed in both severe and moderate cases of infraocclusion, with a cut-off value of 2 mm. As early as 1992, Becker and Karnei-R'em [20] highlighted the relevance of permanent first molars showing vertical undereruption due to infraoccluded primary second molars, suggesting caution in using these teeth as reference points. The current findings support the notion that infraocclusion alone is insufficient to assess the extent of alveolar growth inhibition.

Although various quantitative methods have been proposed to assess alveolar bone in the presence of infraoccluded molars [11, 20, 24, 31, 41], these studies were mostly limited to the mandible. Therefore, the present study applied a classification system applicable to both jaws. However, the assessment of alveolar ridge morphology remains to a certain extent subjective, and the incorporation of both analog and digital panoramic radiographs obtained over an extended time period may have introduced variability related to image quality and interpretation. While vertical ridge patterns have been frequently reported in the literature [16, 42, 43], concave ridge patterns remain poorly documented [15]. A systematic classification, as proposed in this study, has not been described previously. Although spontaneous ridge harmonization after exfoliation has been reported [11, 15], little is known about the long-term development of concave ridge forms.

The timing of onset plays a crucial role. Kurol and Thilander [11] reported progressive infraocclusion during childhood, with an annual increase of 0.8 mm in the maxilla and 0.5 mm in the mandible, peaking between ages 6 and 11. In the present study, no statistically significant correlation between age and infraocclusion severity was observed in the bivariate analysis, whereas the multiple regression analysis demonstrated only a small age-related increase. The discrepancy in magnitude may be attributed to differences in study design, population characteristics, and the retrospective nature of the present investigation, which limits the ability to capture dynamic changes over time. Notably, atypical gingival margins were more frequent in the early mixed dentition, while atypical ridge morphology was more prevalent in the late mixed and permanent dentitions. Latter findings align with observations by Darling and Levers [44], who noted increased infraocclusion in older individuals. The observations suggest that alterations in the gingival margin may serve as a valuable early indicator for the onset of alveolar growth inhibition in infraoccluded primary molars.

This study’s strength lies in its large cohort size compared to similar studies on infraoccluded primary molars, collected over an extended observation period. Nevertheless, as all patients were referred to a university orthodontic department, the presence of selection and referral bias cannot be excluded, which may limit the generalizability of the findings. Additionally, measurements were not feasible in cases where key reference teeth were absent or erupting, resulting in missing data for certain analyses. Moreover, the retrospective design precludes the evaluation of temporal relationships and causal inferences. Consequently, it does not permit conclusions regarding the progression of infraocclusion or associated gingival and alveolar changes over time. Further prospective longitudinal research is necessary, particularly given the potential clinical sequelae, such as the development of lateral open bites.

### Clinical implications

This study demonstrates that infraocclusion alone does not fully characterize the vertical deficiency associated with infraoccluded primary molars. Affected primary molars were significantly associated with an atypical gingival margin. Assessment of the gingival margin using the permanent canine proved more reliable than the primary canine. A gingival margin located ≥ 0.71 mm apical to the reference line connecting the deepest points of the gingival margins of the permanent first molar and permanent canine was identified as a clinically relevant cut-off value. Moreover, an infraocclusion of ≥ 2.0 mm was associated with an increased risk of a vertical or concave alveolar ridge morphology, indicating inhibition of alveolar growth. The inclusion of gingival margin analysis thus provides a more nuanced diagnostic perspective and may serve as an early indicator of developing alveolar ridge abnormalities.

From a clinical perspective, the presence of an alveolar deficiency appears to be a decisive factor in the decision to extract the affected primary molar. In such cases, especially when extraction occurs during early mixed dentition, inserting a space maintainer is highly recommended. Conversely, infraoccluded primary molars without alveolar deficiency signs can be managed with observation. Cases presenting gingival but no alveolar deficiency may require closer clinical monitoring due to the potential risk of progression. When there is no permanent successor, the long-term maintainability of the affected primary molars must be critically evaluated. In general, primary molars that become infraoccluded before puberty are considered to have a poorer prognosis than teeth that become infraoccluded after the pubertal growth spurt, due to the growth potential. Therefore, early extraction combined with orthodontic space closure may be the preferred therapeutic option when indicated.

Overall, this differentiation is clinically relevant as it can help distinguish cases that are likely to remain stable from those at risk of progressing to a concave alveolar ridge morphology and clinically to a lateral open bite. This can support individualized treatment strategies and improved prognostic assessment.

## Conclusions

- The present study identified a significant correlation between infraocclusion, gingival margin position, and alveolar ridge morphology in infraoccluded primary molars.
- Infraocclusion alone was insufficient to accurately characterize the severity of the vertical deficiency at both the gingival and alveolar levels. Atypical gingival margins occurred significantly more often in infraoccluded primary molars compared to healthy controls. Additionally, concave alveolar ridge morphology appeared in both severe and moderate cases of infraocclusion.
- These findings suggest that additional parameters—particularly gingival margin assessment—may enhance early diagnosis and inform treatment planning. Longitudinal studies are needed to evaluate the long-term effects of infraocclusion, especially concerning its potential to restrict vertical alveolar growth.

## Abbreviations

AUC: Area under the curve
CI: Confidence interval
ROC: Receiver operating characteristic
SD: Standard deviation
SEM: Standard error of mean
